# CRISPR-Cas in *Streptococcus pyogenes*

**DOI:** 10.1080/15476286.2019.1582974

**Published:** 2019-03-11

**Authors:** Anaïs Le Rhun, Andrés Escalera-Maurer, Majda Bratovič, Emmanuelle Charpentier

**Affiliations:** a Max Planck Unit for the Science of Pathogens, Berlin, Germany; b Institute for Biology, Humboldt University, Berlin, Germany

**Keywords:** Streptococcus pyogenes, mobile genetic elements, phages, adaptive immune system, CRISPR, Cas9, genome editing

## Abstract

The discovery and characterization of the prokaryotic CRISPR-Cas immune system has led to a revolution in genome editing and engineering technologies. Despite the fact that most applications emerged after the discovery of the type II-A CRISPR-Cas9 system of *Streptococcus pyogenes*, its biological importance in this organism has received little attention. Here, we provide a comprehensive overview of the current knowledge about CRISPR-Cas systems from *S. pyogenes*. We discuss how the interplay between CRISPR-mediated immunity and horizontal gene transfer might have modeled the evolution of this pathogen. We review the current literature about the CRISPR-Cas systems present in *S. pyogenes* (types I-C and II-A), and describe their distinctive biochemical and functional features. Finally, we summarize the main biotechnological applications that have arisen from the discovery of the CRISPR-Cas9 system in *S. pyogenes*.

## Introduction

Bacteria, like humans, are susceptible to viral infections. However, in contrast to human viruses, which are normally detrimental to their host, bacterial viruses, known as bacteriophages (phages), can also be beneficial. Phages can proliferate by producing viral particles and lyse the bacterial cell (lytic cycle). They can also integrate into the bacterial genome and replicate as prophages (lysogenic cycle), thus promoting horizontal gene transfer (HGT).

Through the exchange of genetic material, bacteria acquire new traits that can impact processes such as metabolic pathways, pathogenesis and antibiotic resistance. These exchanges can be mediated through the acquisition of genes from bacterial or phage origin (e.g. phage toxins, toxin-antitoxin systems). Furthermore, phage-mediated DNA transfer may cause profound changes in bacterial gene expression, for example by inactivating host genes or altering the expression of adjacent genes during DNA insertion [1]. In the Gram-positive human pathogen *Streptococcus pyogenes*, the high number of prophages (between 2 and 8, depending on the clinical isolate) that carry virulence-related genes is evidence of the importance of HGT in the evolution of this pathogen [2,3]. Prophages constitute up to 14% of the *S. pyogenes* genome and encode important virulence factors such as streptococcal pyrogenic exotoxins, DNases and the phospholipase SlaA [4]. *S. pyogenes* can cause a wide range of diseases from mild throat and skin infections to life-threatening necrotizing fasciitis. This phenotypic variability can partially be explained by genetic differences among strains that result from HGT. Indeed, phage-derived genes account for most of the inter-strain variability in *S. pyogenes* [5] and encode the main factors triggering T cell response to *S. pyogenes* M1 strain SF370 [6].

In spite of the benefits of HGT, the vast number of phages in the environment implies that the bacteria are under constant phage attack, therefore they need to defend themselves against the risk of lysis or deleterious mutations associated with phage infection. As a consequence, bacteria have evolved a variety of innate and adaptive immune strategies that interfere with each step of the infection process [7–11]. Bacteria can produce extracellular matrix components and/or competitive inhibitors that obstruct phage receptors on the bacterial surface. For example, the hyaluronic acid capsule of *S. pyogenes* is a barrier to the phage A25 which does not encode a hyaluronidase [3,12].

In response to the diverse bacterial immune strategies, phages are constantly evolving, for example, by modifying the affinity of phage proteins for their receptors or by methylating their own DNA to overcome restriction modification systems [13]. This launches a ‘phage versus bacteria’ arms race where both protagonists are tied up in an evolutionary battle for survival.

Apart from the innate immune systems mentioned above, some bacteria also possess an adaptive immune system. The Clustered Regularly Interspaced Short Palindromic Repeats (CRISPR) array, together with the CRISPR-associated (*cas*) genes constitutes, to date, the only known prokaryotic immune system that is able to precisely recognize and target a phage after having ‘memorized’ a specific sequence of its genome [14]. It has also been characterized as one of the few examples of true Lamarckian evolution, as this ‘memory’ can be transmitted to subsequent generations [15]. As observed for innate immunity, phages have evolved mechanisms to counteract this adaptive system, e.g. by using phage-encoded proteins that inhibit the activity of CRISPR-Cas systems, known as anti-CRISPRs [16].

*S. pyogenes* harbors two CRISPR-Cas systems (types I-C and II-A). However, whether there is a trade-off between acquisition of beneficial genes and defense against infection (specifically by CRISPR-Cas systems) is currently unclear for this bacterium. In this review, we discuss the current knowledge on the mechanisms and activity of the CRISPR-Cas systems encoded in *S. pyogenes*, their interplay with phages and their role in the biology of this organism.

## CRISPR-Cas systems

### Mechanism of action

Functional CRISPR-Cas systems are comprised of a CRISPR array and one or multiple *cas* genes transcribed independently or as an operon. The CRISPR array is composed of identical repeats interspaced with short unique sequences called spacers. The spacers originate from mobile genetic elements and function as memory devices that allow recognition of the invaders upon reinfection.

**Figure 1.**
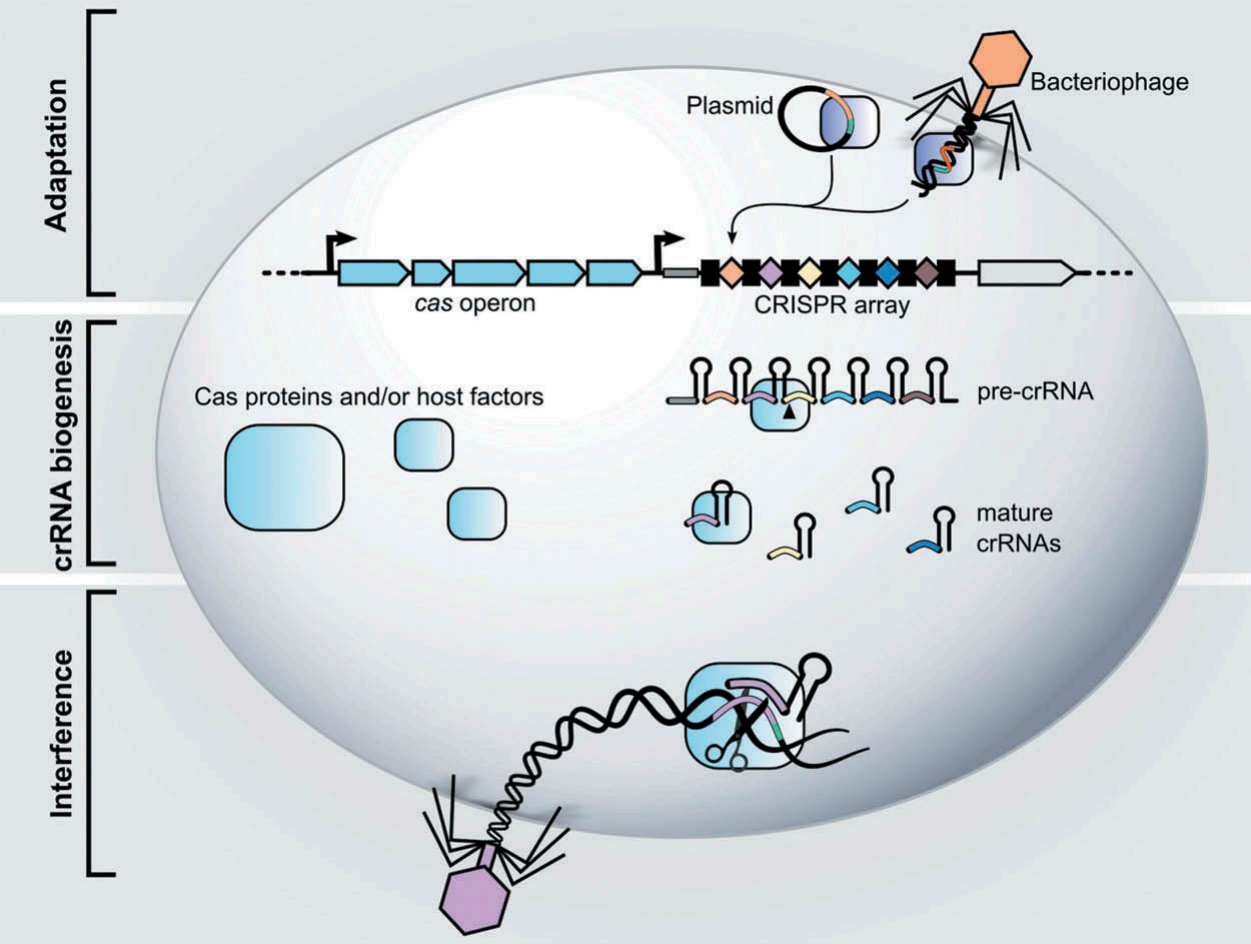
The three stages of CRISPR-Cas adaptive immunity. Stage 1: Adaptation. During this phase, the bacterium incorporates a fragment of the invading phage or plasmid DNA into its genome as a spacer into the CRISPR array (leader: *thick grey line*; repeats: *black boxes*; spacers: *colored diamonds*). This spacer will serve as memory allowing the bacterium to recognize the same threat upon reinfection. After infection, the Cas1-Cas2 complex (*dark blue rectangles*) recognizes the invading DNA and integrates a portion of it (*orange*) into the CRISPR array, giving rise to a new spacer (*orange diamond*). At the same time, a new repeat is generated. Stage 2: crRNA biogenesis. The CRISPR array is transcribed as a long precursor CRISPR RNA (pre-crRNA), which is then processed (as indicated by the *black arrow*) to generate mature CRISPR RNAs (crRNAs), each containing part of a repeat and of a spacer. Processing is mediated either by a Cas nuclease (Class 1 systems, types V and VI) or a host factor (type II). Stage 3: Interference. Either a complex of Cas proteins or a singular Cas protein guided by the mature crRNAs cleave the nucleic acid target in a sequence-specific manner (as indicated by the *scissors*). Upon base paring of the crRNA spacer (*purple*) with the spacer-complementary sequence, known as protospacer (*purple*), a Cas nuclease catalyzes cleavage of the invading DNA leading to its degradation. The PAM sequence, depicted as a *green line*, is a short sequence found next to the protospacer but absent in the CRISPR array, which prevents the array to be cleaved by Cas proteins (autoimmunity). Note that blue rectangles can represent a complex of several Cas proteins or a single Cas nuclease depending on the CRISPR class.

CRISPR-Cas systems act in three stages: (1) adaptation, (2) CRISPR RNA (crRNA) biogenesis and (3) interference, see Figure 1. The adaptation stage involves insertion of a new spacer, derived from the invading genetic material, into the CRISPR array. In the second stage, the CRISPR array is transcribed as a precursor CRISPR RNA (pre-crRNA), which is then processed into mature crRNAs containing a part of the repeat and the spacer. In the final stage, interference, a complex formed by the mature crRNA with single or multiple Cas proteins, recognizes spacer-complementary sequences (protospacers) on the invading nucleic acids and mediates their cleavage. This leads subsequently to the destruction of the foreign genetic material. In some cases, a short protospacer adjacent motif (PAM) sequence located next to the targeted protospacer is necessary for both adaptation and interference stages. In PAM-dependent CRISPR-Cas systems (namely types I, II and V), the PAM sequence, present on the foreign DNA but absent from the CRISPR array, enables self- vs. non-self-discrimination [17–19]. PAM-independent systems have evolved various strategies to avoid self-targeting, such as a protospacer flanking site in some type VI systems [20] or a lack of complementarity between the 5′ repeat handle of the crRNA and the 3′-flanking region of the target RNA for some type III systems [21].

### Classification

To date, approximately 87% of archaeal and 45% of bacterial sequenced genomes found in the CRISPRdb database [22] are predicted to contain CRISPR-Cas systems. Although all CRISPR-Cas systems follow the same general stages and principles, the mechanistic details and machinery involved vary from one system to another. Thus, CRISPR-Cas systems have been classified into two classes, six types and numerous subtypes according to their *cas* gene content, the sequence of the repeats and the organization of the CRISPR loci [23,24]. The two classes differ in the number of Cas proteins involved in interference. While class 1 systems employ multi-subunit Cas protein complexes, in class 2 systems only one Cas effector protein is needed. CRISPR-Cas systems present in *S. pyogenes* belong to type I-C (class 1) and II-A (class 2).

### CRISPR-Cas and horizontal gene transfer

It has been proposed that the presence of CRISPR-Cas systems may be detrimental to the host by preventing the acquisition of potentially advantageous features; for example, studies in *Streptococcus pneumoniae* showed that CRISPR-Cas prevents the acquisition of capsule-encoding genes [25,26]. However, a recent bioinformatics study described that the occurrence of CRISPR-Cas systems (or the number of spacers in a genome) did not correlate with the rate of HGT on evolutionary timescales [27]. This suggests that the presence of CRISPR-Cas systems does not impede the acquisition of new genetic material. This study hypothesizes that since CRISPR-Cas systems are also horizontally transferred, their presence is not indicative of a long-term impact on the HGT-mediated evolution of genomes [27]. In addition, new evidences even suggest that CRISPR-Cas activity could facilitate phage-mediated HGT (transduction) [28].

## CRISPR-Cas loci in *S.* *pyogenes*

### Role of CRISPR-Cas in the evolution of *S. pyogenes*

As explained above, HGT has greatly influenced the evolution of virulence in *S. pyogenes*, but it is less clear how CRISPR-Cas has affected this process (if at all). Indeed, some clinical isolates seem to have lost either the CRISPR array or the complete CRISPR-Cas locus. It was proposed that the absence of CRISPR-Cas could indicate an adaptation to allow acquisition of new virulence factors[1].

In addition to promoting CRISPR-Cas loss, the selective pressure to allow HGT might have affected the activity of CRISPR-Cas systems. It was suggested that the limited spacer content of CRISPR arrays in *S. pyogenes* could reflect a low or null activity of CRISPR [1]. Indeed, while CRISPR arrays of *S. pyogenes* contain between 0 and 7 spacers (depending on the strain and locus) [1,29], other streptococcal species, such as *Streptococcus thermophilus* or *Streptococcus agalactiae*, can have more than 30 spacers in their CRISPR arrays [1,30]. The low acquisition of spacers by *S. pyogenes* might allow the acquisition of diverse virulence factors, explaining the emergence of strain-specific pathogenesis strategies.

Still, there is evidence suggesting that the CRISPR-Cas systems of *S. pyogenes* are active. First, the majority of spacers (27 out of 41) present in all *S. pyogenes* isolates match lysogenic phages found as prophages on the chromosome of other strains. Notably, none of these prophages are located on the same genome as their corresponding spacer, indicating that the CRISPR-Cas systems effectively protected their host [1,29]. In addition, most spacers match known prophages with over 95% identity, and the most conserved spacers are observed only in closely related strains suggesting that these CRISPR-Cas systems were recently active in defense [1]. Indeed, to escape spacer recognition, phages are likely to evolve rapidly. Therefore, the identity between the spacer and its protospacer should decrease over time. Furthermore, active CRISPR-Cas systems tend to diversify by acquiring new spacers and losing/degenerating old ones. For example, in *S. pyogenes* strain SF370, 8 out of 9 spacers showed over 90% identity to sequences within prophages integrated in other strains [18], suggesting that these spacers were recently acquired. This is also supported by the architectural variability of CRISPR loci among clinical isolates from *S. pyogenes* [29], as the order and spacer content of strains is expected to diversify as the strains acquire different spacers.

Overall, it is certain that both HGT and CRISPR immunity have had an important role in *S. pyogenes* evolution. However, further studies are necessary to better understand whether CRISPR-Cas has hindered the acquisition of beneficial genes and its effect on the evolution of *S. pyogenes* virulence.

### CRISPR systems of S. pyogenes

**Figure 2.**
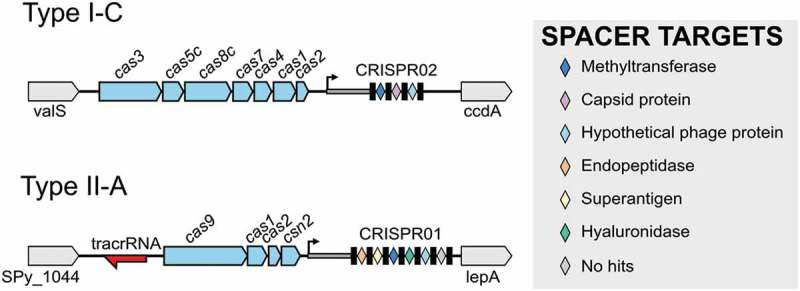
Genomic organization of type I-C and type II-A CRISPR-Cas loci in *S. pyogenes* SF370. The CRISPR array consists of the leader (*thick grey line*), the repeats (*black boxes*), and the spacers (*colored diamonds*). The *cas* genes are represented in *light blue*, flanking genes are shown in *grey* and tracrRNA is shown in *red*. The color of the spacers corresponds to the putative function of their predicted targets (protospacers), as indicated in the legend.

Most of *S. pyogenes* strains contain two CRISPR-Cas loci with different repeat sequences and different sets of *cas* genes [31,32], see Figure 2. The type I-C of *S. pyogenes* SF370 contains seven *cas* genes (*cas3, cas5c, cas8c, cas7, cas4, cas1 a*nd *cas2)* and three CRISPR spacers that target a phage methyltransferase, a capsid protein and a hypothetical protein, respectively. The type II-A of *S. pyogenes* SF370 contains four *cas* genes (*cas9, cas1, cas2 a*nd *csn2)* and six CRISPR spacers targeting a phage endopeptidase, superantigen (speM), a methyltransferase, hyaluronidase, a hypothetical protein and an unknown target [1,29], see Figure 2.

## CRISPR-Cas Adaptation

Adaptation or spacer acquisition is the stage of CRISPR-Cas immunity that enables bacteria to ‘memorize’ a piece of the invaders’ genetic material. It involves several steps: recognition of the invader nucleic acid, selection and processing of the prespacer (spacer sequence, which is about to be integrated), recognition of the CRISPR array and integration of the prespacer into the array as a spacer. The adaptation mechanism is best characterized for type I-E and II-A systems, and has been reviewed elsewhere [33–35]. Here, we focus on types I-C and II-A that are present in *S. pyogenes*.

Two nearly universally conserved Cas proteins are indispensable for adaptation, Cas1 and Cas2 [36,37]. In addition to Cas1 and Cas2, shown to form a complex mediating spacer integration in type I-E of *E. coli* [38], the presence of an A-T rich leader sequence, upstream of the first repeat, is essential for adaptation [37]. Adaptation can be naïve, meaning that a spacer against a phage, which was not encountered prior to infection is integrated into the CRISPR array, or primed, if a spacer targeting the infecting phage is already present in the array. Mutations in the protospacer sequence or in the PAM can prevent interference by CRISPR-Cas, but also stimulate incorporation of new spacers by primed acquisition [39,40].

### Type I-C

Despite being the most commonly occurring CRISPR-Cas systems [23,41], type I-C systems have not been extensively studied. To our knowledge, no experimental data is available for the type I-C system of *S. pyogenes*. Nevertheless, the information arising from recent studies of type I-C in other bacteria may provide insights into the adaptation mechanism of streptococcal type I-C. Type I-C CRISPR-Cas from the pathogenic bacterium *Legionella pneumophila* exhibits robust primed adaptation with the selection of new spacers preferentially from the 5′ region relative to the priming site of the non-complementary strand [42,43].

In addition to Cas1 and Cas2, these systems also encode Cas4 [44,45], which was recently shown to be involved in adaptation [46–48]. Cas4 from *Bacillus halodurans* type I-C system forms a complex with the Cas1 integrase and, in the presence of the Cas1-Cas2 complex, specifically cleaves the 3′ overhangs of prespacers in a PAM-dependent manner. This ensures that only functional spacers get integrated into the CRISPR array, contributing to the accuracy of adaptation [47].

### Type II-A

Studies of type II-A CRISPR-Cas systems from *S. pyogenes* and *S. thermophilus* have shown that, in addition to Cas1 and Cas2, Csn2, Cas9 and *trans*-activating crRNA (tracrRNA) are essential for spacer acquisition in type II-A. Although the catalytic activity of Cas9 is not necessary for adaptation, the Cas9-tracrRNA complex recognizes the PAM on the invading DNA, allowing only functional spacers to be acquired [49,50]. New spacers are preferentially integrated at the leader-proximal end of the CRISPR array. Indeed, five base pairs at the leader-repeat boundary, also known as the leader-anchoring site, are recognized by the Cas1-Cas2 complex and specify the site of integration [51,52]. Interestingly, a study of spacer acquisition upon viral infection showed that spacers are mostly acquired during viral DNA injection, and that free ends of the viral DNA that are first injected into the cell are the preferred source of new spacers [53]. The function of Csn2 remains to be determined.

## CRISPR RNA biogenesis and interference

The pre-crRNA needs to be processed into shorter crRNA fragments to fulfill their function as guide RNAs. The repeats often form secondary hairpin structures that are recognized by a Cas endonuclease. This results in the production of mature crRNAs consisting of a part of the repeat and a unique spacer. During interference, the crRNA forms a complex with one or multiple Cas proteins and is guided by the spacer to the protospacer of an invading nucleic acid via sequence complementarity. This protospacer is then cleaved by a Cas nuclease, preventing plasmid or phage replication.

### Type I-C

In most type I and type III systems, Cas6 is responsible for pre-crRNA maturation. Type I-C does not encode Cas6 but instead the metal-independent endoribonuclease Cas5c (previously called Cas5d) that recognizes and cleaves the hairpin and the 3′ overhang of the pre-crRNA repeat [54]. This cleavage occurs at the 3′-end of the stem-loop structure giving rise to mature crRNAs harboring the repeat-derived 5′ handle and 3′ stem-loop that enclose the targeting spacer sequence [55,56]. Although present in types I-A, I-B and I-E, Cas5 is only directly involved in the maturation process of type I-C. Given the unique functionality of Cas5 in type I-C, and to distinguish it from Cas5 in other types, it was renamed Cas5c [57]. A unique feature of Cas5c is its dual functionality. The protein can process both the pre-crRNA and bind to the crRNA 5′ handle, analogous to the functions of Cas6 and Cas5 from type I-E *E. coli* Cascade (CRISPR-associated complex for antiviral defense), respectively [58].

In *S. pyogenes*, Cas5c specifically processes the pre-crRNA from the type I-C CRISPR but not the one from type II-A [59]. This can be explained by the fact that Cas5c recognizes a hairpin in the pre-crRNA repeats [56,60], which is absent in the unstructured type II-A pre-crRNA. In addition to its endoribonuclease activity, important for pre-crRNA processing, Cas5c possesses a nonspecific double-stranded (ds) DNA binding affinity [59] and promiscuous DNase activity in the presence of divalent metal ions [59,61], suggesting that Cas5c may play a role in multiple stages of CRISPR immunity [61].

The type I-C Cascade is composed of only three Cas proteins, namely Cas5c, Cas7 and Cas8c [54,62]. The structure of *Desulfovibrio vulgaris* type I-C Cascade was determined by cryo-electron microscopy [62]. The complex assembles into a caterpillar-like structure that shows overall similarity to Cas complexes in types I and III. Cas5c remains bound to the 5′ handle of the crRNA after processing, while seven Cas7 subunits wrapped around the crRNA form the backbone of the complex. Cas8c constitutes the ‘belly’ of the complex, analogous to the position of Cas8e and Cas11 (previously called Cse1 and Cse2, respectively) in the type I-E Cascade of *E. coli* [62,63]. Additionally, these subunits appear to be fused in the type I-C Cascade. Cas8c is also involved in PAM recognition and stabilizes the R-loop formed after base pairing between the crRNA and target DNA [62].

Interference in type I systems relies on two components: the Cascade complexes for foreign DNA recognition and the Cas3 nuclease-helicase for cleavage [64,65]. Complete crRNA:DNA base pairing in the type I-E system induces structural rearrangements of the complex and locking of the R-loop, which in turn causes Cas3 recruitment [66]. The molecular details of PAM-recognition and R-loop formation in type I-C require further investigation. Since this system also harbors Cas3, we presume that the interference mechanism is analogous to that of other type I systems.

### Type II-A

**Figure 3.**
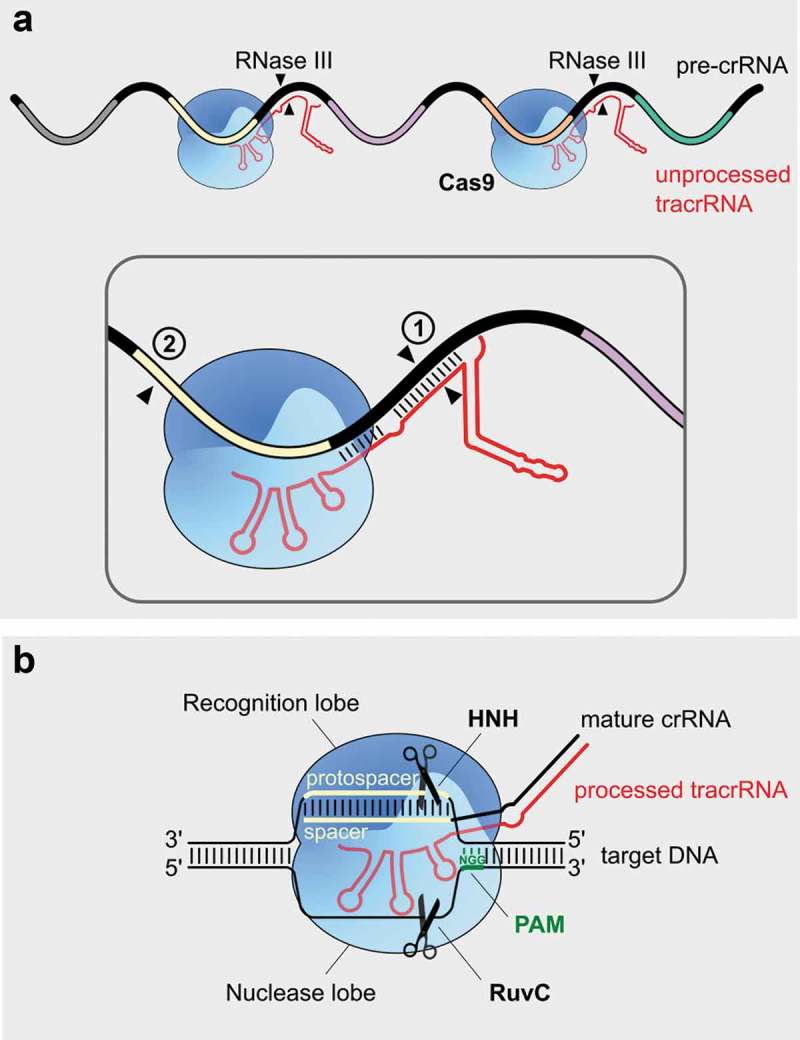
Mechanism of CRISPR-Cas maturation and interference in type II systems. (a). CRISPR RNA maturation by RNase III. The anti-repeat region of tracrRNA (*in red*) base pairs with the repeats of the pre-crRNA (*in black*) in a Cas9-dependent manner (*blue*). ① During the first processing event, the host RNase III recognizes the tracrRNA:crRNA-Cas9 complex and cleaves both tracrRNA and crRNA (*depicted with 2 black triangles*) within the anti-repeat:repeat duplex. ② A second maturation event (*black triangle*) takes place in the 5ʹ of crRNA spacer (*yellow*) by an unknown mechanism, and leads to the production of mature crRNAs. After maturation, the tracrRNA:crRNA duplex remains bound to Cas9. (b). DNA interference by Cas9. The Cas9 endonuclease is then guided by the mature tracrRNA:crRNA duplex to the invading double-stranded DNA (dsDNA). Following Cas9 recognition of the NGG PAM (*in green*), present immediately downstream of the protospacer on the non-complementary strand, the crRNA spacer and its target DNA (both *colored in yellow*) base pair and form an R-loop. This triggers cleavage of the DNA by Cas9 nuclease domains (HNH and RuvC, *light blue*). The HNH domain cleaves the complementary strand, while the RuvC domain cleaves the non-complementary strand, which results in a double strand break on the target DNA. The cleavage takes place three base pairs upstream of the PAM sequence (indicated by the *scissors*) .

Cas9 (previously called *csn1*) and tracrRNA, were shown to be essential for all stages of immunity in the type II-A system [29,49,67]. In *S. pyogenes* SF370, tracrRNA is transcribed from two promoters located in the vicinity of the *cas* operon leading to the expression of two primary transcripts (171 and 89 nts). tracrRNA contains an anti-repeat sequence that base pairs with the pre-crRNA repeats, see Figure 3. This interaction is promoted and stabilized by Cas9 and results in the formation of a stable ribonucleoprotein complex. After formation of the tracrRNA:crRNA-Cas9 complex, both RNAs are co-processed by the host ribonuclease III (RNase III) leading to the formation of a processed tracrRNA (75 nts) and intermediate crRNA (66 nts) forms. This constitutes the first maturation event, followed by an additional processing by unknown factors which gives rise to the 39–42 nt-long mature crRNAs. The resulting mature tracrRNA:crRNA duplex remains bound to Cas9 and it is ready for the interference stage [29]. This maturation event, involving the interaction of two small RNAs, was first described in *S. pyogenes* and then shown to be conserved in all type II systems [68]. tracrRNA and crRNA can be artificially fused into a single-guide RNA (sgRNA), to simplify genome editing applications (see below).

In type II systems, the RNA-guided DNA endonuclease Cas9 mediates cleavage of the invading DNA [67,69,70]. Cas9 has a bilobal structure, which comprises the recognition (REC) lobe, involved in target recognition, and the nuclease (NUC) lobe, involved in target cleavage. The RNA:DNA heteroduplex is positioned in the central channel between the two lobes. The REC lobe contains REC1-3 domains and the arginine-rich bridge helix, while the NUC lobe contains the catalytic HNH and RuvC domains, the guide RNA-interacting Wedge (WED) domain, and the PAM-interacting (PI) domain [71–73]. Binding of the RNA to Cas9 triggers a large conformational change that enables the protein to scan for PAM sequences on the foreign DNA via three dimensional diffusion [74–77]. Upon recognition of the PAM sequence (NGG for *S. pyogenes* Cas9), the double-stranded DNA melts, which enables the crRNA spacer to probe for complementarity with the target DNA [71]. If there is sufficient complementarity, the spacer base pairs to the complementary DNA strand and the non-complementary strand is displaced, forming an R-loop structure [67,78]. R-loop formation is directional and proceeds from the PAM-proximal towards the PAM-distal part of the target [77,78]. Both the PAM recognition and spacer-protospacer base pairing in the 10–12 nt PAM-proximal seed sequence are essential requirements for Cas9 endonuclease activity leading to a blunt double-strand break of the target, three base pairs upstream of the PAM [67,70].

The HNH and RuvC endonuclease domains cleave the complementary and non-complementary strands of the protospacer, respectively [67]. Upon target DNA binding, the HNH domain undergoes conformational transitions from an inactive to an active state, which controls cleavage activity. The transition to the active state is dependent on divalent cations and complementarity in the PAM-distal part of the target [79]. The REC3 domain of Cas9 was shown to sense the spacer:protospacer complementarity, and control the docking of the HNH domain to the cleavage site [80]. Cleavage by the RuvC domain is allosterically regulated by the conformational activation of the HNH domain. This ensures simultaneous cleavage of the target DNA by both endonuclease domains [81].

## Harnessing type II CRISPR-Cas for biotechnology

One of the most common approaches used by researchers to investigate the role of a specific gene is analyzing the effect that its inactivation or overexpression has on an organism. Therefore, advancements in biological sciences have been tightly linked to the development of tools that allow facile and accurate genetic manipulation. The use of genome-engineering techniques enables modulation of a particular gene (or a set of genes), while maintaining the context relatively unchanged. Furthermore, precise modification of the genetic content and/or its expression is an attractive strategy for treating a great variety of genetic diseases, viral infections and cancers.

**Figure 4.**
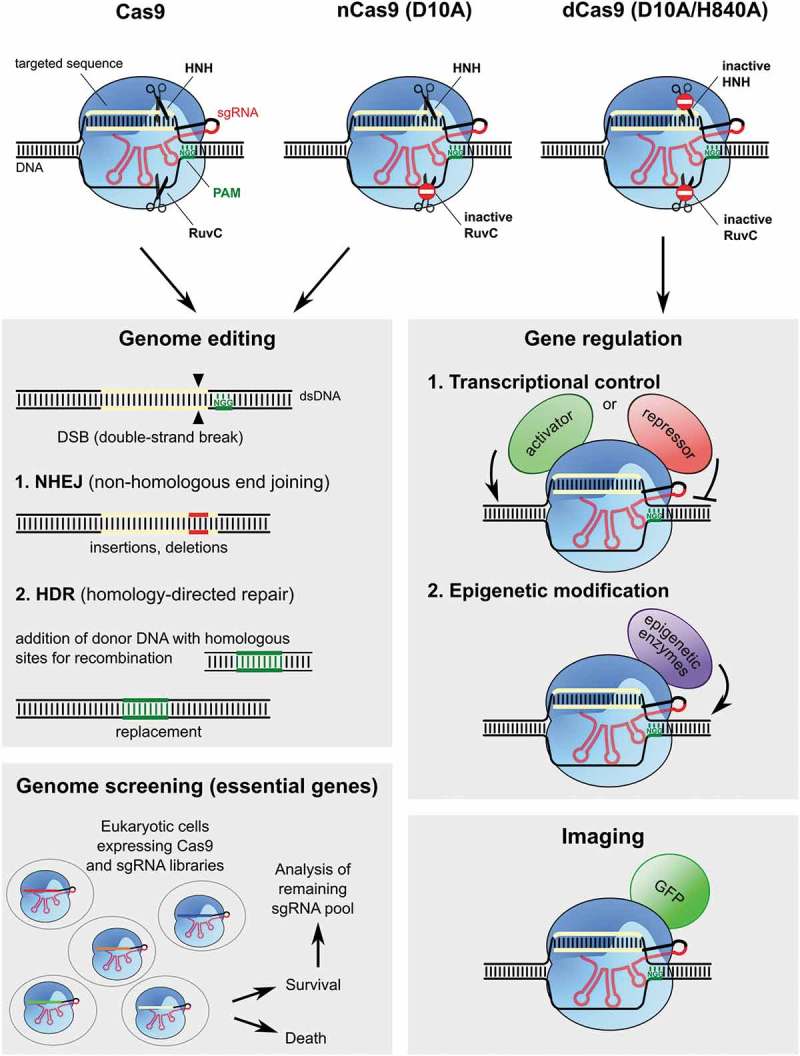
Applications of RNA-guided Cas9. Cas9 (*in blue*) from *S. pyogenes* and the guide RNAs have been engineered and repurposed for a variety of biotechnological applications. The construction of the sgRNA (*in red*), which mimics the tracRNA:crRNA mature duplex, allows targeting of Cas9 to the desired sequence simply by changing the sequence that corresponds to the spacer (*yellow*). Using the wild type (WT) Cas9 allows generating double strand breaks (DSBs) in virtually any desired sequence (as long as it is adjacent to the NGG PAM, *in green*). If, instead of the WT Cas9, a mutant in one of the nuclease domains is used (nickase Cas9 or nCas9), a nick will be generated on the target DNA. A D10A substitution inactivates the RuvC domain while a H840A mutation inactivates the HNH domain, in both cases without affecting its affinity for DNA (domain inactivation is represented by the *stop sign on the scissors*). Using paired nCas9 (two D10A nCas) to create two nicks instead of a DSB improves the specificity. Inactivating both nuclease domains generates a dead Cas9 (dCas9), which specifically binds to the target DNA without affecting its integrity. **Genome editing**. DSBs or nicks produced by Cas9 at specific sites can be repaired by the non-homologous end-joining (NHEJ) or the homology-directed repair (HDR) systems. While the NHEJ pathway generates an insertion and/or deletion, HDR leads to the replacement of the specific sequence with a sequence from the DNA donor template. Targeting Cas9 to a gene of interest can be used to generate deletion mutants or to replace one gene with, for example, a different allele. **Gene regulation**. Catalytically inactive dCas9 can be fused to various effector domains like: 1. A transcriptional activation domain (*green*) or a transcriptional repression domain (*red*) to control the transcription of target genes or 2. epigenetic effectors (*in purple*) to regulate epigenetic modifications (e.g. DNA methylation, histone modifications) of the target loci. **Imaging**. dCas9 can also be fused with the Green Fluorescent Protein (GFP in *green*) to allow the visualization of specific loci in cells. **Genome screening**. Eukaryotic cells expressing Cas9 are first infected with a sgRNA library. Each cell will contain one sgRNA targeting a specific sequence (each color corresponds to a different spacer). The cells are then treated with a particular drug or compound and sgRNAs of the survivor cells are sequenced and compared to the ones of the initial population. The cells where a sgRNA targets an essential gene for survival, under the studied condition, are killed by the specific Cas9 DNA cleavage. This leads to the identification of genes that are indispensable for survival in the response to a certain challenge.

Permanent and complete inhibition of gene expression can be achieved by inducing DNA-double strand breaks (DSBs), see Figure 4. DSBs are repaired naturally by non-homologous end-joining (NHEJ) or homology-directed repair (HDR) pathways, which may lead to frameshift mutations or gene replacement, respectively.

The major challenge for taking advantage of the natural repair mechanisms is to develop a tool that allows the generation of DSBs efficiently and specifically on the sequence of interest. As a result, three main programmable genome-editing tools have been generated: zinc-finger nucleases (ZFNs) [82], transcription activator-like effector nucleases (TALENs) [83,84], and RNA-guided Cas proteins. ZFNs and TALENs require extensive protein design and optimization in order to target the desired sequence, which has limited their applicability. In contrast, Cas9 can be easily reprogrammed to target a desired sequence simply by designing a sgRNA that is complementary to the DNA region of interest [67]. After the discovery that Cas9 from *S. pyogenes* could be directed to cleave almost any DNA sequence that is adjacent to a PAM (NGG for *S. pyogenes* Cas9), its potential as a genome-editing tool became clear [67]. Shortly after being demonstrated *in vitro*, the ability of Cas9 to cause specific DSBs on target DNA sequences was first demonstrated in human and mouse cells [85–87], and then in a plethora of other organisms [88–90]. In addition to providing a useful tool for generating animal and plant models, RNA-guided Cas9 can be used to screen for genes that are essential for cell survival under certain conditions or for specific drug targets [91–95].

Although its value in genome engineering is beyond doubt, some questions remain to be addressed before Cas9 can be used in the clinic, such as Cas9 specificity (*i.e*. the propensity to induce off-targets). Namely, Cas9 has a certain level of tolerance for mismatches between the target and the guide sequence, especially when they are located outside the PAM-proximal seed sequence (8–12 nt) [67,85,87,96,97]. Off-target cleavage rates by Cas9 vary depending on a number of factors, including Cas9 expression level [98], target sequence [99] and quantification methods. Several approaches have been applied to reduce potential unwanted mutations [80,100–106].

By introducing inactivating substitutions in either Cas9 nuclease domain, Cas9 can be converted into a nickase (nCas9) that produces single strand breaks (SSB). When two nCas9 are directed by two sgRNAs targeting adjacent sequences, both DNA strands are cleaved. Because nicks are normally repaired without introducing mutations, only regions where nicks are produced in close proximity will be mutated, reducing off-target effects drastically [107].

Similarly, the catalytically inactive Cas9 mutant (dCas9) can be fused to a monomer of the FokI enzyme, which is only active as a dimer, and directed to adjacent sequences in order to promote the DSBs. This reduces off-targets because dCas9-FokI molecules will only cause DSBs when bound next to another dCas9-Fok molecule, which is unlikely to occur as a result of unspecific interactions [101,105]. Modulating the activity of *S. pyogenes* Cas9 using anti-CRISPR proteins can also reduce off-targets by ensuring it is only active when needed and in the desired tissues [108,109].

Furthermore, to improve Cas9 specificity on the protein level, both structure-guided and random mutagenesis approaches have been employed, leading to the development of *S. pyogenes* Cas9 variants with enhanced specificity, namely eSpCas9 [104], SpCas9-HF1 [102], HypaCas9 [80], and evoCas9 [100]. Since off-target activity varies depending on the target of interest, Cas9 variants with enhanced specificity might abolish off-target cleavage for some sites while showing significant off-target activity for others. Therefore, there is still a substantial interest in further improving Cas9 specificity, while retaining on-target activity, as well as understanding the mechanisms that control specificity in detail.

dCas9 may also be used for reversible regulation of gene expression. If targeted to transcription factor binding sites or promoters, dCas9 can directly compete with RNA polymerase binding or transcription initiation. Alternatively, dCas9 can be directly fused to transcriptional activators or repressors, which can be directed to the gene of interest [110]. dCas9 has also been fused to histone modification enzymes (e.g. methylases) to manipulate epigenetic modifications) [111–113]. Furthermore, when fused to fluorescent markers, such as the green fluorescent protein, dCas9 can be used to visualize a specific DNA locus inside the cell [114,115]. Fusion of dCas9 with a cytidine deaminase [116] or transfer RNA adenosine deaminase [117] enables changes in a single base pair without the need for DSBs. This method, called base-editing, offers some potential for novel strategies to treat human diseases caused by point mutations in the DNA [118].

This and other applications of Cas9 for genome editing and modulation have been the topic of several reviews [88,90,110,118–122].

## Conclusion and perspectives

The discovery and characterization of RNA-programmable Cas9 emerged from basic research on the type II CRISPR-Cas system from *S. pyogenes* and has provided a new biotechnological tool for genome editing and modification that is revolutionizing the field of molecular biology. Although the technology is now widely used for many different applications, limitations such as Cas9 off-target activity and the the inherent constraints of cellular repair mechanisms still remain to be solved. Nevertheless, the CRISPR-Cas9 technology, derived from the human pathogen *S. pyogenes*, now holds a promising potential to develop novel strategies to fight and cure many diseases.

Despite the attention that CRISPR has received and the relevance of *S. pyogenes* as a human pathogen, the biological role of CRISPR in this organism, including potential regulatory functions as well as its impact on horizontal gene transfer, is yet to be understood. It was proposed that the presence and activity of CRISPR-Cas systems are contributing factors in *S. pyogenes* ecology and evolution. For example, it is possible that CRISPR activity limits the spread of advantageous features [25,26]. Yet, this view is in apparent contradiction with the observation that prophages and seemingly active CRISPR-Cas systems often co-occur on the same chromosome. One explanation proposed by Gophna *et al* [27] is that CRISPR-Cas systems act as mobile genetic elements that protect bacteria at the population level without affecting the genetic composition of the host in the long term. Nevertheless, more studies are necessary to determine if this hypothesis explains the distribution of CRISPR-Cas systems and mobile genetic elements across *S. pyogenes* strains. The increasing availability of sequenced genomes will facilitate studying the interplay between defense systems and HGT, and its impact on bacterial evolution, leading to a better understanding of epidemiological phenomena such as the transfer of virulence factors by phages.
